# Care providers’ view of the barriers in providing care for adolescents with emotional and behavioral problems

**DOI:** 10.3389/fpsyg.2024.1302004

**Published:** 2024-12-19

**Authors:** Katerina Paclikova, Zuzana Dankulincova Veselska, Andrea Madarasova Geckova, Peter Tavel, Danielle E. M. C. Jansen, Jitse P. van Dijk, Sijmen A. Reijneveld

**Affiliations:** ^1^Olomouc University Social Health Institute, Palacky University in Olomouc, Olomouc, Czechia; ^2^Department of Community and Occupational Medicine, University Medical Center Groningen, University of Groningen, Groningen, Netherlands; ^3^Department of Health Psychology and Research Methodology, Faculty of Medicine, PJ Safarik University in Kosice, Kosice, Slovakia; ^4^Faculty of Social and Economic Sciences, Institute of Applied Psychology, Comenius University Bratislava, Bratislava, Slovakia; ^5^Department of General Practice and Elderly Care Medicine, University Medical Center Groningen, University of Groningen, Groningen, Netherlands

**Keywords:** emotional and behavioral problems, adolescence, care providers’ perspective, psychosocial care, barriers in psychosocial care

## Abstract

**Objectives:**

Emotional and behavioral problems (EBP) during adolescence are a major public health issue due to its high prevalence and long-lasting consequences. The knowledge of the barriers in providing psychosocial care can be a starting point for subsequent efficiency improvement. Therefore, the aim of this study is to assess which barriers do care providers experience while providing psychosocial care for adolescents with EBP.

**Methods:**

We performed a qualitative assessment that was embedded in the Slovak Care4Youth study mapping the system of care provided for adolescents with EBP. We conducted 25 semi-structured individual and group interviews with a total of 49 care providers from 17 institutions that provided preventive counselling, social, and mental healthcare for adolescents with EBP. We focused on the care provider’s perception of barriers in providing care for these adolescents. The interviews were audiotaped and transcribed verbatim. We coded the data using the consensual qualitative research approach in combination with conventional content analysis.

**Results:**

We found that care providers experienced several barriers in providing care for adolescents with EBP which relate to six themes—legislative framework and legislative changes; financing of the care system; coordination of care; workforce development, education, methodical guidance and supervision; personnel and institutional capacities; and administrative burden.

**Conclusion:**

Addressing these barriers within the care system from the “front line” perspective provides clues to efficiently improve the psychosocial care for adolescents with EBP. According to the care providers, the organization and financing of the care system requires adaptation; the burden of the care providers should be reduced; and care providers require quality practical education, training, and methodological guidance.

## Introduction

Emotional and behavioral problems (EBP) are a major public health issue in adolescents because of their long-lasting consequences (de et al., 2022; Jaspers et al., 2012) and high prevalence. In Europe, approximately 15.5% (from 5.7 to 36.7%) of adolescents are suffering from EBP. The rates per country, from lower to higher, for Europe are – Denmark (5.7%), England (11.2%), Lithuania (13.1%), Austria (23.9%), and Turkey (36.7%) (Sacco et al., 2022). In Slovakia, recently a study on the health behavior in school children (HBSC) reported that 21–22% of adolescent girls had depression and 30–32% of them had anxiety (HBSC, 2023). A recent, pilot study in the Czech Republic nation showed that around 35% of the adolescents had depression or anxiety (NUDZ, 2023).

Emotional and behavioral problems are defined as “behaviors or emotions that deviate so much from the norm that they interfere with the child’s own growth and development and/or the lives of others” (Cooper, 2011, p. 71–72). Emotional problems include depression, withdrawal, social phobia, specific phobias, anxiety, post-traumatic stress disorder, obsessive-compulsive disorder, poor self-esteem, as well as feelings of inferiority, self-consciousness, shyness, hypersensitivity, and somatic complaints or eating disorders. Behavioral problems include defiance, impulsivity, disruptiveness, aggression, antisocial behavior, and overactivity as well as problems with attention and self-regulation, such as temper tantrums, substance abuse, and bullying (Achenbach et al., 1991; Bornstein et al., 2013; Ogundele, 2018). If these problems are not identified early and adolescents do not receive adequate professional care, they experience worse functioning in various life domains, and these problems may worsen into more severe emotional and behavioral disorders persisting into adulthood (Verhage et al., 2021; Ormel et al., 2017, Costello and Maughan, 2015, Kretschmer et al., 2014, Raudino et al., 2012, Dougherty et al., 2015, Luby et al., 2014, Stringaris et al., 2014, National Institute for Health and Care Excellence, 2013).

In Slovakia, adolescents with EBP can receive care from various institutions, depending on various factors, such as the seriousness of the problem, which person recognized the problem in the adolescent, which professional is easy to reach and trusted by the parents, and whether the family is willing to cooperate or not (Mackova, 2022). The system of psychosocial care for adolescents in Slovakia comprises of three main types of care that often overlap: preventive counselling, social, and mental healthcare. The preventive counselling branch focuses primarily on problems related to the school environment, it mainly deals with diagnostics and counselling, and it falls under the Ministry of Education, Science, Research, and Sports. The social branch is covered by the Ministry of Labour, Social Affairs, and Family. It includes services for families in difficult life situations and the protection of the rights and interests of minors. Finally, mental healthcare falls under the Ministry of Health and includes primary care for mental health and psychiatric care provided through outpatient clinics or hospitals (Dankulincova et al., 2020).

Not all adolescents with EBP receive adequate care (Paclikova et al., 2020). The care for the adolescents who experienced EBP is often unequally distributed, with some adolescents not receiving any care (George et al., 2018; Nanninga et al., 2018; Vasileva and Petermann, 2017; Langer et al., 2015; Jansen et al., 2013), which could be partially explained by adolescents having sub-threshold symptoms (Pace et al., 2023), and some of them receiving care without identified EBP (Paclikova et al., 2020). By a system of psychosocial care, we mean all institutions and professionals working there that come in contact with adolescents with EBP, their working procedures, as well as existing relationships, connections, and possibilities of cooperation between individual institutions (Dankulincova et al., 2020). Research shows that there are issues in searching, accessing, and using psychosocial care by parents and adolescents (George et al., 2024; Nanninga et al., 2018; Fridman et al., 2017; Garland et al., 2017; Nanninga et al., 2016; Nanninga et al., 2015; Reijneveld et al., 2014; Oruche et al., 2014; Martinez and Lau, 2011). Since Slovak adolescents cannot make decisions about their health on their own without the consent of their parents, the parents play a crucial role in the whole process of entering into and providing psychosocial care for their children (Mackova, 2022).

Most of the literature with regard to the barriers in providing care for adolescents with EBP is from the perspective of the parents and/or the adolescents themselves. These studies show barriers such as stigmatization, lack of information about the services, the beliefs about the services and the trust of care providers, the accessibility of services, the demographic characteristics of the family, family relationships, and racial/ethnic minority (Powell et al., 2021; Lu et al., 2021; Bains, 2014, Plaistow et al., 2014). Evidence is limited on the barriers in providing care for adolescents with EBP from the perspective of the care providers (Paton and Hiscock, 2019; Cole et al., 2016). Their voice is often not heard although they are likely to have important information for the policymakers that can help to improve the structure of the care system and the care provided for adolescents with EBP.

Theoretical models of access and use of psychosocial care along with its associated barriers mostly differentiate three main levels — society, the care system itself, and provider/client (Andersen, 1968; Durbin et al., 2006; Kringos et al., 2010; Levesque et al., 2013). In the present study, we focus on the barriers regarding the care system. Previous research addressing this level, shows these barriers to first regard limitations at a governance level, such as a lack of coordinated services and regional variation in service availability, which make access to care problematic (Dankulincova et al., 2020; Paton and Hiscock, 2019; Levesque et al., 2013) and inadequate organization of the system of care, which leads to parallel care and multiple entries of adolescents to care which can be intrusive and counterproductive (Cole et al., 2016; Dankulincova et al., 2020). Moreover, some research show limitations regarding the financing of the care system, such as low salaries or adverse working conditions influencing the number of professionals and resulting in limited efficiency of care provided (Kidia et al., 2017; Mackova, 2022). Other barriers include a gap in the transition to adult care and a limited continuity of care (Paton and Hiscock, 2019). In addition, a high administrative burden and lack of available personnel are the limitations to optimal provision of care (Paton and Hiscock, 2019; Cole et al., 2016; Oruche et al., 2014; Pedrini et al., 2012; Pfefferle, 2007) as well as insufficient methodical guidance and education perceived by care providers (Hetrick et al., 2011; Paton and Hiscock, 2019; Kidia et al., 2017; Dankulincova et al., 2020).

Previous research on the barriers to psychosocial care provided to adolescents with EBP focuses only on one particular type of care, e.g., social care or healthcare or on only some barriers in the system of care, thereby bringing fragmented evidence. Our study adds by addressing the full range of the barriers perceived by care providers in the whole care system — preventive counselling, social and mental healthcare—the types of care which tend to overlap in Slovakia. Thus, it can provide clues for efficiency improvement. Therefore, our aim is to assess what barriers are experienced by the providers of psychosocial care for adolescents with EBP at the care system level.

## Methods

### Design of the study

We conducted an exploratory qualitative analysis as a part of the Slovak Care4Youth (C4Y) study by mapping the Slovak system of psychosocial care provided for the adolescents with EBP and their characteristics. This study was realized using a combination of the consensual qualitative research (CQR) methodology (Hill et al., 1997) and conventional content analysis (Hsieh and Shannon, 2005). We chose the conventional content analysis since it is suitable for studies which aim to describe a phenomenon and leads to categories and names of categories that arise from the data itself instead of using preconceived categories. The knowledge generated from the conventional content analysis is based on the participants’ unique perspectives grounded in the actual data (Hsieh and Shannon, 2005). We also included the elements of a consensual approach to introduce the multiple perspectives of individual team members participating in the process of the analysis of qualitative data. The CQR methodology involves the diversity of experiences and opinions of each member of the research team in the research process, and their ability to reach conclusions regarding their assumptions (Hill et al., 1997; Hill and Knox, 2021). This limited the potential impact of the subjective perspectives of the researchers, as these had to achieve consensus related to the codes used for the analyzed data. Its design was approved by the Ethics Committee of the Medical Faculty at P. J. Safarik University in Kosice (protocol 2 N/2015).

### Study setting, sampling, and participants

Our sample consisted of professionals providing care for adolescents with EBP. The system of psychosocial care in Slovakia comprises of three main types of care: preventive counselling, social, and mental healthcare. The preventive counselling branch focuses primarily on problems related to the school environment, mainly dealing with the diagnostics and counselling, and it falls under the Ministry of Education, Science, Research, and Sports. The social branch is covered by the Ministry of Labour, Social Affairs and Family, that includes services for families in difficult life situations and the protection of the rights and interests of minors. Finally, mental healthcare falls under the Ministry of Health and includes primary care for mental health and psychiatric care provided through outpatient clinics or hospitals (Dankulincova et al., 2020). Thus, three Ministries are involved in providing care to adolescents with EBP.

To obtain our sample of providers, we used a two-step sampling approach to reach a representative sample of all types of institutions, preventive counselling, social or mental healthcare in Slovakia. Firstly, we identified and chose representatives of all types of institutions in preventive counselling, social, and mental healthcare in the Košice region, Slovakia. We contacted the selected institutions, and all the 17 institutions that we approached agreed to participate in the study. Secondly, within each institution, we asked all the care providers working with adolescents to participate in the study and all of them agreed. We provided them with the information about the study, informed them about the voluntary and anonymous basis of the participation, and provided them with informed consent forms to read and sign if they decided to participate in the study.

We conducted 25 individual and group interviews with the care providers in the period between May 2017 and November 2018, involving one to four providers per interview (49 care providers were interviewed in total). We performed 18 group and 7 individual interviews, with the type of interview depending on the type of institution. Group interviews were performed in institutions with multidisciplinary teams. We took care that all respondents could express their opinions as much as possible in the group interviews similar to that in the individual interviews by explicitly paying attention to this topic and structuring the interviews such that each participant got room to express their own opinions. Since some of the care providers were the head or the chief of the institution at the same time, they were interviewed separately apart from the employees in order to create a space for open expression of attitudes which was otherwise potentially hindered by the power imbalance.

### Procedure and measures

We performed semi-structured interviews by mapping the system of care for adolescents with EBP from the perspective of the professionals. We conducted interviews with the care providers from the psychosocial care institutions who provide care to children and adolescents with mental health problems. Additionally, to better guide our respondents during interviews, we asked them at the beginning of the interview to focus on their answers in aspects relevant to their work with adolescents with EBP. The interviews lasted approximately 60–120 min and took place in the institutions where the care providers worked. The interviews were conducted by one to three interviewers (the principal investigator conducted all the interviews, and the others alternated) in the Slovak language and were recorded. The research team consisted of researchers with a background in psychology and social work at different levels of their academic careers with various experiences from their previous work in the system of care. All interviewers were trained researchers, and they conducted interviews using a semi-structured topic guide consisting of several questions mapping the system of care. One question specifically oriented on the perceived barriers by care providers: “*What are the barriers that do not allow you to work with clients/families according to your liking?.”* This question was embedded in a wider set of questions about different aspects of the care provided by the institution, including questions on the competencies of providers, set-up of the work with clients, the methods they used in their work and their theoretical background, cooperation with other institutions, perceived barriers and facilitators of care, and the provider’s suggestions about what might help to improve the care for adolescents (for the full guide, see Supplementary Material).

### Data handling, analysis, and reporting

We handled the data by transcribing the interviews verbatim in the Slovak language and the transcriptions were checked to ensure accuracy. Subsequently, we coded the data using the CQR methodology to provide a basis for the conventional content analysis (Hsieh and Shannon, 2005). Three teams of coders (one team per branch of care) were involved. Each team consisted of the principal investigator, senior researchers, and a PhD student. Two main coders (the principal investigator and a PhD student) read and coded all the interviews across all three branches of the system of care. Other senior researchers were chosen based on their experience with the appropriate type of system of care. All coders had been trained in CQR. We started by reading and familiarizing ourselves with the data which was followed by data-driven production of initial codes. Each team member read the transcripts of the interviews and created codes for parts of the interviews individually. Thereafter, the team members met and shared their codes with the aim of achieving consensus. In the case of differing opinions, the discussion continued until a consensus was reached. We used the MAXQDA software as a standard platform for data analyses (VERBI Software, 2021) for the coding process.

Once the initial coding was performed, we conducted a conventional content analysis (Hsieh and Shannon, 2005) with clustering codes with regard to the reported barriers in the system of care as perceived by the care providers categorizing them into subthemes and themes. To ascertain this, the team members read all the codes and sorted them into groups of subthemes and overarching themes based on the topic they were covering. This creation of subthemes and themes were done separately for each of the three types of care. Later on, the principal investigator with senior researchers and the PhD student combined the subthemes and themes from all three types of care and looked for overlaps and differences between them. However, our research team did not find any major discrepancies in the barriers described by the interviewed care providers in the three main types of care. In cases when we found some smaller differences, the team members searched for a theme that would cover all the nuances in order to conclude with overarching theme for all three types of care.

## Results

### Characteristics of the sample

The sample consisted of 49 care providers from 17 institutions that provided preventive counselling, social, and mental healthcare for adolescents with EBP. They had the following professional background: 20 psychologists, 18 social workers, 4 child psychiatrists, 4 teachers, 1 educational counsellor, 1 pedagogue for adolescents with special needs and 1 nurse. Some of the care providers were also the head or the chief of the institution where they worked. The care providers in our sample worked with adolescents from different backgrounds (socio-economic status of the family, parental education, family composition etc.) including but not limited to only those from low-risk communities (for a detailed overview of the sample, see Table 1).

**Table 1:** Characteristics of the sample by type of care, participants’ profession, gender, and years of practice in the current position.

Interview	Type of care	Participants’ profession	Gender	Years of practice in current position
1	Preventive counselling care	R1: Teacher R2: Teacher	R1: Female R2: Female	R1: 17 R2: 19
2	Preventive counselling care	R1: Educational counsellor	R1: Female	R1: 8
3	Preventive counselling care	R1: Chief of the institution/Psychologist R2: Psychologist	R1: Female R2: Female	R1: 11 R2: 3
4	Preventive counselling care	R1: Chief of the institution/Pedagogue for special needs R2: Psychologist	R1: Female R2 Female	R1: 12 R2: 3
5	Preventive counselling care	R1: Chief of the institution R2: Psychologist R3: Psychologist	R1: Female R2: Female R3: Female	R1: 10 R2: 8 R3: 5
6	Preventive counselling care	R1: Psychologist	R1: Female	R1: 2
7	Preventive counselling care	R1: Teacher/Pedagogue for special needs R2: Social worker R3: Social worker	R1: Female R2: Female R3: Female	R1: 11 R2: 1 R3: 5
8	Preventive counselling care	R1: Chief of the institution/Teacher	R1: Male	R1: 19
9	Social care	R1: Chief of the institution/Psychologist R2: Psychologist	R1: Female R2: Female	R1: 15 R2: 1
10	Social care	R1: Chief of the institution/Social worker R2: Social worker	R1: Female R2: Male	R1: 15 R2: 5
11	Social care	R1: Psychologist R2: Psychologist R3: Social worker	R1: Female R2: Female R3: Female	R1: - R2: - R3: -
12	Social care	R1: Social worker R2: Social worker	R1: Female R2: Male	R1: 1 R2: 1
13	Social care	R1: Psychologist R2: Social worker	R1: Female R2: Female	R1: 2 R2: 2
14	Social care	R1: Psychologist R2: Social worker	R1: Female R2: Female	R1: 1 R2: 1
15	Social care	R1: Psychologist R2: Psychologist	R1: Female R2: Female	R1: 24 R2: 12
16	Social care	R1: Social worker R2: Social worker	R1: Female R2: Female	R1: 7 R2: 5
17	Social care	R1: Chief of the institution/Social worker R2: Chief of the institution/Social worker	R1: Female R2: Male	R1: 23 R2: 19
18	Social care	R1: Psychologist R2: Social worker	R1: Female R2: Female	R1: 8 R2: 5
19	Mental healthcare	R1: Psychologist R2: Psychologist	R1: Female R2: Female	R1: 10 R2: 10
20	Mental healthcare	R1: Chief of the institution/Social worker	R1: Female	R1: 20
21	Mental healthcare	R1: Chief of the institution/Psychiatrist	R1: Female	R1: 3
22	Mental healthcare	R1: Psychologist R2: Psychiatrist R3: Nurse R4: Social worker	R1: Female R2: Female R3: Female R4: Female	R1: 2 R2: 21 R3: 14 R4: 12
23	Mental healthcare	R1: Psychologist	R1: Female	R1: 4
24	Mental healthcare	R1: Psychiatrist	R1: Female	R1: 8
25	Mental healthcare	R1: Chief of the institution/Psychiatrist R2: Psychologist	R1: Female R2: Female	R1: 3 R2: 1

### Barriers experienced by care providers—themes and subthemes

We identified the barriers that the participants experienced while providing care to the adolescents with EBP and categorized them into six themes: 1. Legislative framework and legislative changes, 2. Financing of the care system, 3. Coordination of care, 4. Workforce development, education, methodical guidance, and supervision, 5. Personnel and institutional capacities, 6. Administrative burden. We provided a detailed description of the identified themes and subthemes (in italics) with the selected participant quotations below (Table 2).

**Table 2:** Identified themes and subthemes regarding the barriers in the system of care for adolescents with emotional and behavioral problems.

**Themes/Subthemes**
**Legislative framework and legislative changes**
legislative framework limits and/or restricts the possibilities within the organization of the system of care
legislative framework limits and/or restricts the competencies of care providers
legislative framework limits and/or restricts the institutional and/or personnel capacities within the system of care
frequent legislative changes and insecurities about the implementation of legislative changes
management of institutions by several ministries with unclear responsibility
**Financing of the care system**
insufficient and/or lack of finances from ministries for institutions and low wages for care providers
time-limited finances for institutions through projects with lack of continuity
limits from insurance companies
**Coordination of care**
parallel, duplicate, and/or repeated care provided by various care providers
insufficient and/or lacking interdisciplinary collaboration between care providers
**Workforce development, education, methodical guidance, and supervision**
insufficient and/or lacking high-quality and practical education
insufficient and/or lacking methodical guidance
insufficient and/or lacking supervision
**Personnel and institutional capacities**
lack of care providers
lack of specialized facilities
insufficient material and spatial conditions
**Administrative burden and overload**
high administrative burden and bureaucracy
lack of time and capacity
time pressure
overload of tasks

### Legislative framework and legislative changes

Regarding the first theme, the participants described several barriers influencing the provision of care for adolescents with EBP in the system of care. They mentioned the *legislative framework as limiting their work and possibilities within the organization of the system of care*. Legislative framework defines which care can and cannot be provided as well as the amount of care, which participants perceived as limiting. They mentioned that in the case of state institutions, the law precisely defines what a given provider or institution can do within the scope of provided care, what services, to what extent, for how long, under what conditions and for which clients. As a result, the participants mentioned that they were unable to provide care that they felt would be needed or effective.

*“You must follow the law. You cannot go above it, but not below it either in provision of care…”* (chief of the institution, preventive counselling care).

The participants also mentioned that the *legislative framework is limiting their competencies*. According to the participants, any initiative in favor of the client outside the competence defined by the legislation is not permissible.

*“A state institution can only do what the law allows you to do, so as not to abuse the power. Precisely strictly defined, if it is stated there [in the law], if it is not [stated in the law], I am sorry, you cannot do it because you are a state institution.”* (chief of the institution/social worker, social care).

Another barrier perceived by the participants was the *legislative framework limiting personnel capacities within the system of care*. Some of the participants stated that ministries were limiting the number of staffs in the facilities although they felt that more people would be needed in order to fulfil the demand in the system of care.

*“Unfortunately, we do not have the staff and the ministry does not allow us [to increase] the numbers [of employees].”* (teacher/pedagogue for special needs, preventive counselling care).

A part of this theme was also that the participants mentioned *frequent legislative changes and their insecurities about the implementation of the legislative changes* as one of the barriers to the provision of care in the system of care. Some of them felt uncertain on whether they would be able to adapt to these changes as an organization or whether some organizational and personnel changes would be needed.

*“And here we get [accreditation from the Ministry of Labour], but at the same time we also have school programs and they are subject to the Ministry of Education, but there have been various changes, so now we are dealing with it there, how it will turn out in the end, whether we will simply apply for accreditation because we used to have accreditation at the Ministry of Education, now it has changed and now I don't know how it will be…”.* (psychologist, social care).

Others felt that the changes often came from the top level and did not consider the actual needs of the care providers who were meeting the clients on a daily basis and were able to see what the system might need in order to be more effective. Regarding that, some of the participants reported that:

They were aware that even in such ideal conditions the change could take years. As a result of this process, the participants described their frustration and resignation that sometimes they saw no possibility of change within the system of care. Finally, the participants mentioned a barrier that is: the *management of institutions by several ministries with unclear responsibility*.

“*You need to work in the capital city to be able to lobby for a needed change*”. (social worker, social care)

*R:* …we belong to the Ministry of Interior in terms of property and personnel, i.e., professional employees, but we carry out activities and are financed by the Ministry of Education.

I: So, you fall under two ministries?

*R: Yes, which is no longer a very happy solution, but that's how they decided, that's how they came up with it. It's not a very happy, not a very happy solution.”* (chief of the institution, preventive counselling care).

In the words of some of the participants, the management of the institutions by several ministries eventually led to not taking responsibility for the institution by any of the ministries.

### Financing of the care system

With regard to the second theme, the participants also mentioned that the financing of the care system is a barrier. *Insufficient finances from the ministries for the institutions* were perceived as one of the barriers that related to *low wages* in care professionals which as a result often led the institutions to need multi-source financing in order to enable the institution to operate and provide care.

*“They will write the budget in January. I can already see that December is not covered at all. In October, we only have half of the salary, let's do it. We are not even talking about operating costs. […] So, we have such a budget. Now last week we were. So, when I saw it, I turned pale, we lack 60,000 Euros. For completely normal, for completely normal operational things.”* (chief of the institution/ pedagogue for special needs, preventive counselling care).

Some of the participants mentioned *time-limited finances for institutions through projects with lack of continuity* as barriers. As a result of this form of financing, job positions covered by those projects are time-restricted, the participants feel the instability in work and continuity of care cannot be secured.

*“Well, for the most part, the projects were implemented, but as soon as those projects ended, those assistants lost their jobs in 80% of cases, including professional employees who worked at the school…”* (psychologist, preventive counselling care).

Participants in mental healthcare pointed out that *limits from insurance companies* play an important role in financing too by limiting the frequency and amount of care via provided funding only if specific diagnoses are given to the adolescents with EBP.

*Papers [with limits] from insurance companies simply arrive, which we have to accept, take into account.”* (psychologist, mental healthcare).

### Coordination of care

Participants also reported barriers related to coordination of care which is our third identified theme. As one of the barriers within this theme, the participants mentioned *parallel, duplicate and/or repeated care provided by* var*ious care providers*. They explained that this parallel care causes adolescents to wander through the system of care from one care provider to another and as a result to experience repeated entry and actions from various care providers.

*“That’s the amount of people who go to that family and in front of them that child or that parent has to explain something. They don’t like it anymore. Well, I wouldn’t enjoy it either. To have so many people involved and to talk to so many, in front of so many people, about your things and problems.”* (social worker, social care).

Another barrier mentioned by participants regarding coordination of care was the *insufficient interdisciplinary collaboration with other care providers*. Participants talked about the lack of information from other care providers and find the information exchange insufficient and rather lengthy. They perceived feedback from other care providers on what actions were done with clients and what care was provided for them as essential for efficient care. However, some of the participants were not able to get it from other care providers and were dissatisfied with the inability to provide ongoing care. If collaboration was present between care providers some participants stated that recommendations for working with clients, they received from other institutions were sometimes perceived as unrealistic as they did not have the conditions or competencies for the implementation of such recommendations (e.g., unfeasible implementation of recommendations for working with adolescents in schools). In other cases, recommendations for working with clients from other providers were completely lacking. Some of the participants mentioned that they do not collaborate with other care providers due to previous bad experiences in collaboration.

*“The cooperation doesn’t really work that well. For example, I was also initially interested, when I was a recent graduate, in communicating with other psychologists about how they solve similar problems. So that I might actually get into that practice somehow.”* (psychologist, preventive counselling care).

### Workforce development, education, methodical guidance and supervision

Within the fourth identified theme, a first barrier mentioned by participants was *insufficient high-quality and practical education*. Participants stated that available education does not reflect their needs for gaining practical skills for pursuing professional development, it is mostly theoretical and often lacks quality.

*“… they push us teachers into education… but, they need to make such an education that would really help the teacher. How to work with this child [with emotional and behavioural problems]. Because teachers are willing to learn…. Because, we can’t help ourselves when a child throws a chair against the wall and runs away from the classroom…”* (educational counsellor, preventive counselling care).

They mentioned that their preceding university education does not adequately prepare them for professional practice. Participants also complained about lacking affordable education because they often must pay for it themselves, as it is not financially supported by their employer. Another barrier regarded *insufficient methodical guidance*. Participants explained they perceived the quality of care provided and procedures and techniques used by care providers differed by region. They reported this to be probably due to insufficient methodical guidance of care providers and inconsistent system in each type of care.

*“They will call a meeting. We will have a meeting where they will explain it [new regulation]. But, again, someone is reading it. The same thing that is on paper… I can read it. What I need is a transfer into practice. Let me know how it works in practice. What can I do, what competencies do I have, how should I do it?”* (social workers, social care).

They also pointed out they are seriously *lacking supervision* and effective interdisciplinary meetings.

*„Because before I came here, they didn’t have a psychologist for about half a year, and then the teachers started giving me such feedback that it was great, that finally, someone is actually paying attention to the children, that you can see the changes. Then I was so happy, because in the beginning I really didn’t get any feedback and I, a graduate, didn’t know if I was doing things well. “*(psychologist, preventive counselling care).

### Personnel and institutional capacities

As a part of the fifth theme, participants mentioned a *shortage of care providers* in the system of care. They talked about high employee turnover in the system of care for adolescents with EBP due to unattractiveness of the profession, insufficient payment, job insecurity, and overload.

*“I think there are few experts for those children, for those families. There are few psychologists and they deal with it adequately, but I don’t want to offend anyone, but as quickly as possible.”* (psychiatrist, mental healthcare).

Participants viewed as a barrier also a *shortage of specialized facilities* and of capacities in existing facilities which are insufficient for exceeding number of adolescents with EBP who are in need of professional care.

*“A [number of] facilities of this type is very inadequate… we are always full, we are often overcrowded, and the waiting time is long. It's just not like we get a referral today and the child can come in a week. There is also such a possibility, sometimes it happens that the child has to wait for several weeks.”* (nurse, mental healthcare).

Participants further mentioned *insufficient spatial and material conditions* in the institutions. Most of the participants stated that lack of finances for institutions resulted in the lack of facilities, equipment, and special tools needed for work.

*“… for a year we can’t get money for the emergency situation on the ground floor. It is in a desolate state. When you entered, you could smell the aroma from the non-functioning toilets and so on, and we simply cannot get the money for a year, more than a year.”* (chief of the institution/pedagogue for special needs, preventive counselling care).

Participants expressed their opinion that the above-mentioned barriers lead to long waiting periods in the system of care.

### Administrative burden

One of the highlighted barriers mentioned by participants in the sixth theme regarded the *high administrative burden* as a result of excessive *bureaucracy*.

*“I think that, for example, it would be improved, or I would perceive it that way, if so, much paper documentation was not needed at the expense of the work with the given client. Let’s face it, I take it as a systemic flaw, but I don’t expect us to eliminate it.”* (psychiatrist, mental healthcare).

Participants felt an administrative burden regarding their everyday activities as overwhelming and taking away the time needed to work with their clients.

“Diagnosis … it is difficult, because the child does not do much, therefore it takes a long time. The interview with the parent takes a long time. And it takes twice as long to process the conclusion to be useful. That’s the whole day, or four hours, five. But that’s just one client and that’s not enough for a day. Plus, if we want to do a group, such as psychotherapy groups, speech therapy groups, then you need rooms, you need people, you need time.” (chief of the institution/pedagogue for special needs, preventive counselling care).

They also felt a pressure for quick results in connection with a *lack of time and capacity.*

*“I do not have time for that at all, so I really leave this [psychotherapy] to the psychologist, to whom I will either recommend the child, or they already have one and will continue.”* (psychiatrist, mental healthcare).

and overall *time pressure* resulting in *overload* as a barrier for the provision of effective care.

*“If we had perhaps fewer of those cases, there would be more people and that person would have more time for the family. He is able to pay more attention to the family, also to talk with the parents, and with the child. For example, we cannot afford to have some clients here for an hour and a half, or just two hours, because others are just getting upset behind the door.”* (social worker, social care).

## Discussion

The aim of this study is to assess which barriers are experienced by care providers in psychosocial care for adolescents with EBP in the level of the care system. We found six themes regarding barriers experienced by the participants while providing care for adolescents with EBP—legislative framework and legislative changes, financing of the care system, coordination of care, workforce development, education, methodological guidance and supervision, personnel and institutional capacities, and administrative burden.

### Legislative framework and legislative changes

We found that the care providers perceived the legislative framework to be limiting their work and the possibilities within the organization of the care system, as well as their own competencies and personnel capacities. The participants also perceived insecurities regarding the legislative changes and management by several ministries with unclear responsibilities. These issues were also found in other studies (Paton and Hiscock, 2019; Cole et al., 2016; Oruche et al., 2014; Pfefferle, 2007). As the care system usually involves several ministries, the cooperation that is needed for the successful organization of the care system is considered rather problematic (Dankulincova et al., 2020; Paton and Hiscock, 2019). In addition, an organization of the care system often does not reflect “the reality” and needs to be articulated by the providers themselves. Thus, care providers might perceive little or no control over the legislative conditions as these can only be changed through political processes (Dankulincova et al., 2020). Increasing the competencies of the care providers together with improving their working conditions are crucial in enhancing the care system for adolescents with EBP as repeatedly stated in the previous qualitative research by the care providers (Dankulincova et al., 2020; Paton and Hiscock, 2019; Kidia et al., 2017; Thomée et al., 2016).

### Financing of the care system

Subsequently, we found that care providers perceived insufficient finances for institutions from the ministries to be related to low wages, and they mentioned that time-limited finances for institutions through projects with a lack of continuity is a barrier. As mentioned, in the previous studies, if the organization of care is not provided with precise and continued allocation of financial resources that are required, then effective provision of care becomes limited (Dankulincova et al., 2020; Kidia et al., 2017). Another barrier perceived by the care providers includes limits from the insurance companies. This was found to be one of the economic barriers to mental healthcare in previous research as well (e.g., Flisher et al., 1997). Some of the care providers might consider those limits as unacceptable since they had to accept the limits from the insurance companies that are in contrast to their beliefs about the best interest of the clients (Dankulincova et al., 2020; Mackova, 2022). As a consequence, these insurance limits might lead some of the care providers to take up the position of freelancers (those being paid for care provided directly by clients instead of care being covered by insurance companies). Overall, it creates the dilemma mentioned by Mackova (2022) of deciding whether to exclude clients who are unable to pay for the treatment by themselves in exchange for gaining freedom in other factors of care, or providing care fully paid by the insurance company in exchange for having to accept limits set by these companies on the number of clients/sessions per client and in the case of exceeding these limits, to working for free.

### Coordination of care

Care providers reported parallel, duplicate, and/or repeated care provided by various care providers as a barrier. They also pointed out insufficient interdisciplinary collaboration with other care providers. Previous research reported that (e.g., Bramesfeld et al., 2012), the coordination of care via interdisciplinary cooperation was considered as a basic requirement in the provision of effective care. Although the importance of coordination of care is well understood, the experience of cooperation with other care providers might range from very active to very passive (Mackova, 2022). It could relate to the unclear or completely missing definition of the roles and competencies between specific care providers, which created a certain barrier for cooperation. As reported by Suter et al. (2009), good cooperation between different professionals is based on clear definition and understanding of the roles and competencies of all the involved care providers. Lack of coordination might also be explained on the basis of the role of parents in the care. In cases where the parents did not agree, the care provider could not contact or cooperate with other professionals. Such non-cooperation from parents was perceived by care providers as a potential barrier for the effective care as well (Mackova et al., 2022; Dankulincova et al., 2020).

### Workforce development, education, methodological guidance, and supervision

Care providers described lack of high quality and practical education as another barrier. They also reported insufficient methodological guidance and supervision. Previous research shows lack of standardized methodological guidance and recommendations for practice as a barrier by care providers (Hetrick et al., 2011; Paton and Hiscock, 2019; Pedrini et al., 2012; Pfefferle, 2007). Similar opinions were also expressed by care providers in previous qualitative research (Dankulincova et al., 2020; Paton and Hiscock, 2019; Kidia et al., 2017; Thomée et al., 2016) that further education, training, supervision, and overall workforce development is crucial for the provision of optimal care. Following the recommendations of Glisson (2002), quality education and training for care providers are one of the determinants in providing efficacious care.

### Personnel and institutional capacities

We found that care providers perceived a shortage of manpower in the care system and in specialized facilities, including insufficient spatial and material conditions in the institutions. Our findings are in line with previous research (Dankulincova et al., 2020; Kidia et al., 2017) suggesting that inadequate capacities limit the accessibility of care (Levesque et al., 2013) in that the absence of care offered in the client’s place of residence creates a barrier in the client’s access to essential help. Inadequate capacity can also affect the quality of care provided, as workers do not have the space for intensive, continuous, and long-term work with clients, or the whole family, which in many cases would be appropriate. Care providers express dissatisfaction with the spatial conditions and material equipment available to them, which limit their work and make it unpleasant for them and for clients as well (Dankulincova et al., 2020).

### Administrative burden

Care providers identified a significant barrier in the form of substantial administrative burden, characterized by bureaucracy, lack of time and capacity, resulting in overloading work. Previous research brought similar findings (Cole et al., 2016; Hetrick et al., 2011; Oruche et al., 2014; Paton and Hiscock, 2019; Pedrini et al., 2012; Pfefferle, 2007). Administrative burden was, in a previous study, attributed to very few personnel and institutional capacities. As a consequence, it ultimately affects the limited time available to work with the client. This may reduce the quality of the work and time that the provider has to devote to one client. On the contrary, there is a selection of clients and not everyone gets access to services that would be optimal from the point of view of solving the adolescent’s problems (Dankulincova et al., 2020).

### Barriers in the overall care system

Previously discussed themes regarding the identified barriers within the care system for adolescents with EBP match the “building blocks” as described by the World Health Organization (WHO, 2010) in their framework. It describes the health systems in terms of six core “building blocks”—service delivery, health workforce, health information systems, access to essential medicines, financing, and leadership/governance. The barriers can be found within each of these areas. Although the WHO framework is oriented on healthcare, in our study, we were able to also identify barriers in similar areas like preventive counselling care as well as in social care, suggesting that all parts of the system that provides care for adolescents with EBP struggle with similar limitations. Overall, according to the statements from the care providers on the barriers within the care system for adolescents with EBP, it seems that the existing system is struggling with being limited and insufficient, thereby hindering its effectiveness.

### Strengths and limitations

The strength of our study lies in its application of the consensual qualitative research approach, which incorporates different perspectives from several researchers on the topic of study. Additionally, we successfully arranged a sample of care providers from all types of institutions within the care system in Slovakia. Furthermore, care providers in our sample selection have worked with adolescents with different backgrounds (socio-economic status of the family, parental education, family composition etc.) including individuals from both low-risk and high-risk communities. Such variability increases the potential generalizability of our results. The first limitation of our qualitative research (and qualitative research in general) relates to the expectations and biases including the subjective view of the researchers, which might influence the analysis. However, we believe that this was reduced by engaging the perspectives of different researchers and using a consensus during coding and analysis. The second limitation of our study could be with regard to the combination of individual and group interviews during data collection. However, the researcher conducting the interviews ensured that all respondents expressed their opinions as much as possible in group interviews as it should happen in individual interviews. The third limitation of this study is that we may not have achieved the maximum saturation of professionals in terms of the number of interviews conducted. However, the sample selection was designed to reach a heterogeneous sample representative of the main types of psychosocial care providers, and we achieved that. Furthermore, during data collection, we reached a point of saturation, in which no new themes occurred.

### Implications

Our findings have several implications for practice. Firstly, the organization and financing of the care system need to be adapted to enhance the quality of the care provided. These adaptations should relate to the themes that we identified and should be based on the experience of the professionals working with the adolescents in the front line. According to the care providers, one of the biggest problems is the lack of interdepartmental cooperation, as the institutions are managed by different ministries and thus, they are subject to different, often changing, legal regulations with different ways of management and funding. The needed adaptations must relate to a system vision, both being socially and politically important. Secondly, the burden of care providers should be reduced. One of the ways is to eliminate redundant bureaucracy. Additionally, the bureaucratic burden for the care providers resulting from the current laws only prolongs and slows down the entire care process. In order to be able to provide the most effective help in solving problems, it is important to first of all ensure adequate and especially continuous funding, which would help to improve working conditions leading to material security and adequate financing of professions that correspond to the difficulty of the work performed. Furthermore, the attractiveness of the work and its working conditions should be improved, so that more personnel and more capacity of care providers become available. Finally, care providers need quality practical education, training, and standardized methodological guidance to improve the quality of care provided and support their competencies.

Policymakers should consider these findings as an overview of the system-level barriers within the care system for adolescents with EBP which might be beneficial for further improvements and proposals for changes in the care provided. Changes in the care system are often made without “hearing the voice of professionals,” yet their point of view brings valuable and necessary information about what is not working and what needs attention to reach adequate changes.

Our study also has implications for further research. Incorporating the views of parents as well as adolescents on the same barriers that care providers experienced might complete the overall picture of this topic. The perspective of all involved parties is a fundamental element for efficient changes in the care system. Further research should focus on the possible changes and improvements, and the already functional and positive aspects of the psychosocial care system from the perspective of clients and care providers.

## Conclusion

The knowledge and understanding of the care system as a whole is crucial to effectively set up processes in the provision of care for adolescents with EBP. Only by identifying the shortcomings and needs of all the care takers (in this case, care providers) can the recommendations and solutions be proposed that would increase the effectiveness of the care system. The care providers experience several barriers with regard to providing psychosocial care for adolescents with EBP in that the organizations and financing of the care system need adaptation; the burden of the care providers should be reduced; and care providers need quality practical education, training, and methodological guidance. Understanding the complex picture of barriers within the care system from the “front line,” i.e., as seen through the eyes of care providers, can provide clues to efficiently improve psychosocial care provided for adolescents with EBP.

## Data Availability

The raw data supporting the conclusions of this article will be made available by the authors, without undue reservation.
