# Supplementary material for: Care providers’ view of the barriers in providing care for adolescents with emotional and behavioral problems
Source: Front Psychol. 2024 Dec 19;15:1302004. doi: 10.3389/fpsyg.2024.1302004 (PMC11693648; doi:10.3389/fpsyg.2024.1302004)
Supplement: Supplementary file 1 [file Data_Sheet_1.PDF]

Semi-structured interview with care providers mapping the system of care

Legislative and financial framework

(1) How is your institution providing care to adolescents with EBP legislatively framed?

(2) Whom is your institution legally subjected to?

(3) Which laws apply to the operation of your institution?

(4) What is your institution's license, who grants it to you, and with what criteria?

(5) How is the purpose of your institution being declared?

(6) What competencies does your institution have?

(7) What services does your institution provide?

(8) What is the personal composition of your institution?

(9) What is the source of your institution's funding, on what performance?

Working with a clients

(1) What types of clients are appearing in your institution? Is it possible to divide them into groups? What is the typical case?

(2) What is usually the reason for your client / family to enter the care in your institution?

(3) Where do the client / family come from, where do they go, what other institutions are offering care at the same time?

(4) How do you determine the type of client / family problem in your care?

(5) What kind of care is provided to clients / families (consultation, counselling, therapy)?

(6) How do you choose which form of care will be provided to the client / family?

(7) How long has your institution been given care to the client / family?

(8) How often do you meet your client / family in the care provided?

(9) How do you evaluate the success of the form of care provided?

(10) What should be the optimal care provided to your client / family?

(11) What is the end of your institution's provision of care to the client / family?

(12) What is the reason for ending the care on behalf of your institution? What is the reason for ending care on behalf of your client / family?

(13) What are the barriers that prevent you from doing your work with clients / families according to your ideas?

(14) What changes would be needed in the legislation or in the overall care system to make your work with clients / families more effective?
